# Hemostatic Balance in Pediatric Acute Liver Failure: Epidemiology of Bleeding and Thrombosis, Physiology, and Current Strategies

**DOI:** 10.3389/fped.2020.618119

**Published:** 2020-12-23

**Authors:** Yonca Bulut, Anil Sapru, Gavin D. Roach

**Affiliations:** ^1^Department of Pediatrics, Division of Critical Care, David Geffen School of Medicine, University of California, Los Angeles, Los Angeles, CA, United States; ^2^Division of Pediatric Hematology-Oncology, David Geffen School of Medicine, University of California, Los Angeles, Los Angeles, CA, United States

**Keywords:** PALF, hemostasis, coagulopathy, children, critical care, transfusion, thrombosis, liver failure

## Abstract

Pediatric Acute Liver Failure (PALF) is a rapidly progressive clinical syndrome encountered in the pediatric ICU which may rapidly progress to multi-organ dysfunction, and on occasion to life threatening cerebral edema and hemorrhage. Pediatric Acute Liver Failure is defined as severe acute hepatic dysfunction accompanied by encephalopathy and liver-based coagulopathy defined as prolongation of International Normalized Ratio (INR) >1.5. However, coagulopathy in PALF is complex and warrants a deeper understanding of the hemostatic balance in acute liver failure. Although an INR value of >1.5 is accepted as the evidence of coagulopathy and has historically been viewed as a prognostic factor of PALF, it may not accurately reflect the bleeding risk in PALF since it only measures procoagulant factors. Paradoxically, despite the prolongation of INR, bleeding risk is lower than expected (around 5%). This is due to “rebalanced hemostasis” due to concurrent changes in procoagulant, anticoagulant and fibrinolytic systems. Since the liver is involved in both procoagulant (Factors II, V, IX, XI, and fibrinogen) and anticoagulant (Protein C, Protein S, and antithrombin) protein synthesis, PALF results in “rebalanced hemostasis” or even may shift toward a hypercoagulable state. In addition to rebalanced coagulation there is altered platelet production due to decreased thrombopoietin production by liver, increased von Willebrand factor from low grade endothelial cell activation, and hyperfibrinolysis and dysfibrinogenemia from altered synthetic liver dysfunction. All these alterations contribute to the multifactorial nature of coagulopathy in PALF. Over exuberant use of prophylactic blood products in patients with PALF may contribute to morbidities such as fluid overload, transfusion-associated lung injury, and increased thrombosis risk. It is essential to use caution when using INR values for plasma and factor administration. In this review we will summarize the complexity of coagulation in PALF, explore “rebalanced hemostasis,” and discuss the limitations of current coagulation tests. We will also review strategies to accurately diagnose the coagulopathy of PALF and targeted therapies.

## Introduction

Pediatric acute liver failure (PALF) is a life-threatening illness, characterized by the sudden onset of coagulopathy, thrombocytopenia, and systemic inflammation that may ultimately result in dreaded complications such as cerebral edema and hemorrhage. The liver is responsible for the synthesis of a majority of blood coagulation factors and plays a central role in the hemostatic system. As a result of decreased coagulation factor synthesis, acute liver failure patients were historically believed to be “autoanticoagulated” and protected from thrombosis. However, this belief has been challenged as the understanding of the coagulopathy in liver failure has evolved. In acute liver failure, there is a parallel reduction of anti-coagulant factors along with pro-coagulant factors and this leaves the patient in a rebalanced coagulation state that may sometimes favor thrombosis (1–7). Consequently, thrombotic events are more common than bleeding complications in patients with acute liver failure (2–4, 8–11). Clinically significant bleeding is rare, in part due to the absence of esophageal varices and portal hypertension, which are the major risk factors for bleeding in chronic liver failure. Recent data shows that in acute liver failure the spontaneous significant bleeding rate is <5%, the spontaneous intracranial bleeding rate is <1% and the risk does not correlate with INR values (12).

Since PALF is defined as severe acute hepatic dysfunction with prolongation of International Normalized Ratio (INR) >1.5 with encephalopathy, close monitoring of INR and correction of elevated levels occur frequently (13). Although the INR value is followed as a prognostic tool it is important to recognize the limitation of this classic test for evaluating the coagulopathy of liver disease. The prothrombin time (PT) and INR are measures of pro-coagulant factors (or lack thereof) and do not take into consideration changes in anti-coagulant factors, platelets, and other hemostatic changes. As such, the PT and INR are poor predictors of bleeding risk. It is essential to be cognizant of the risk of over-transfusion and tipping this delicate balance toward hemorrhage or thrombosis for an arbitrary INR number (14). Transfusion of any blood component has been associated with increased mortality, increased need for liver transplantation and reduction in graft survival (15). Transfusion-related immunomodulation (TRIM), transfusion-related acute lung injury (TRALI), transfusion-associated circulatory overload (TACO), hemolytic reactions, and infection risk are some of the potential reasons for transfusion-related poor outcomes (16). This review will discuss the pathophysiology of “rebalanced hemostasis” in pediatric acute liver failure, diagnostic challenges and therapeutic considerations.

## Rebalanced Hemostasis

The hemostatic system consists of three major arms. Primary hemostasis involves formation of the primary platelet plug on the injured vascular endothelium. Secondary hemostasis involves the cascade of coagulation factors leading to formation of the fibrin clot. Fibrinolysis follows primary and secondary hemostasis; and involves a process that removes the fibrin clot and prevents uncontrolled thrombosis (17, 18).

In acute liver failure, all three of these arms of hemostasis are affected. The delicate balance between prevention of bleeding and excessive clot formation is impacted by the concomitant changes in pro- and anti-hemostatic factors in PALF. These changes are a result of decreased hepatic synthesis of pro- and anti-coagulation factors, thrombocytopenia, elevated von Willebrand factor (vWF), reduced a disintegrin and metalloproteinase with a thrombospondin type 1 motif, member 13 (ADAMTS-13), reduced vitamin-K dependent carboxylation of pro- and anti-coagulant factors, and consumptive coagulopathy (10, 19).

### Primary Hemostasis

**Figure 1:**
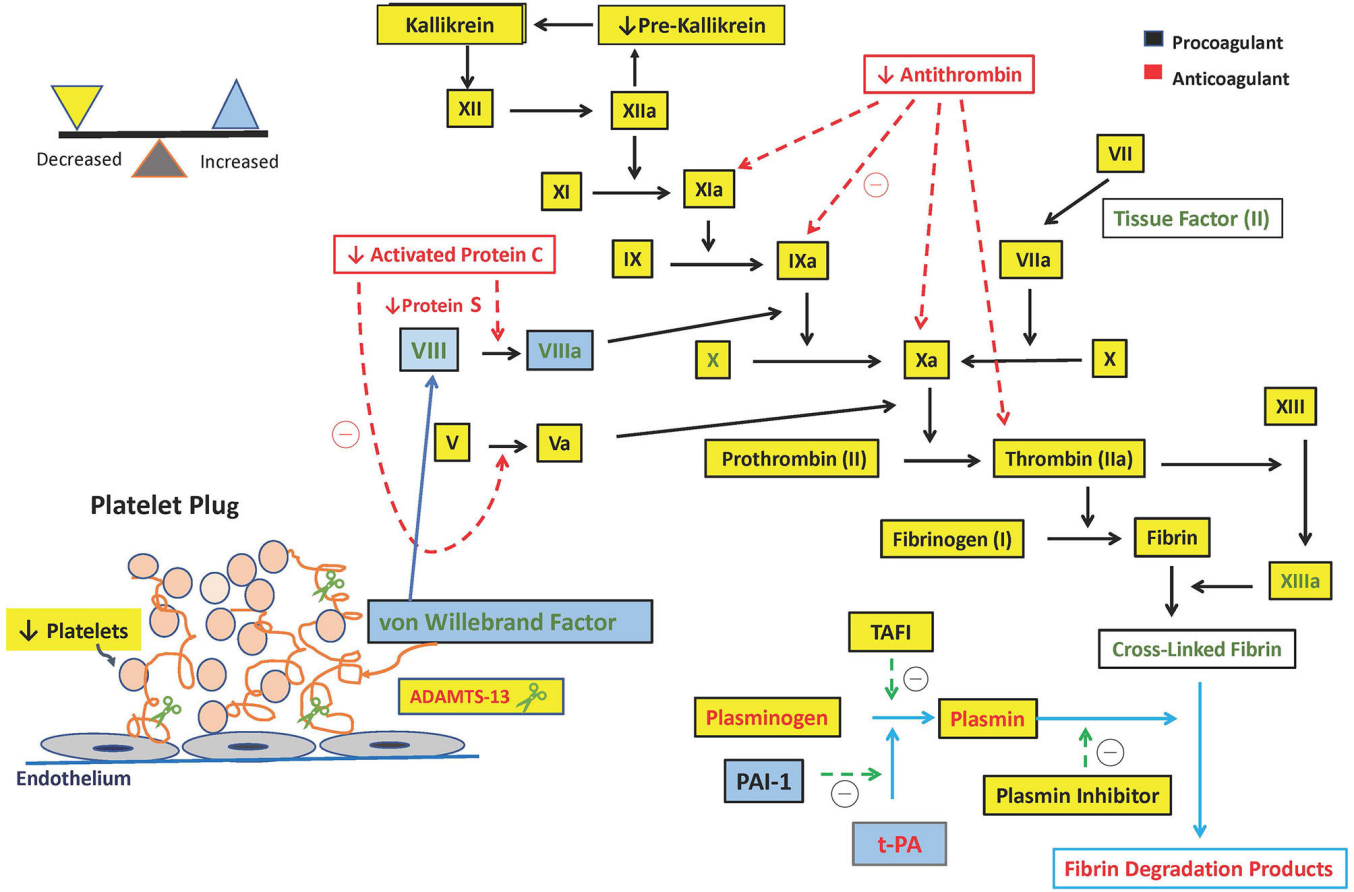
Hemostatic alterations in pediatric acute liver failure. ADAMTS-13, a disintegrin and metalloproteinase with a thrombospondin type 1 motif, member 13; t-PA, tissue plasminogen activator; TAFI, thrombin-activatable fibrinolysis inhibitor; PAI-1, plasminogen activator inhibitor type 1; u-PA, Urokinase-type Plasminogen Activator.

Platelets are responsible for the initial primary hemostatic response by adhering to the damaged blood vessels and promoting aggregation and clot formation with the aid of the endothelial-derived protein vWF. They are also responsible for facilitating the Tissue Factor (TF) and factor VII complex formation, which catalyzes the activation of factor X and formation of “priming thrombin” (19) (Figure 1).

Both platelet number and function are reduced in PALF, although the changes are less pronounced compared to chronic liver failure (2, 9). Thrombopoietin (TPO) is a glycoprotein hormone produced primarily in the liver that regulates the production of platelets in bone marrow by stimulating megakaryocytes. In children with acute liver failure there are reductions in TPO levels as evidenced by reduced serum TPO and hepatic TPO mRNA levels (20). Moreover, increased consumption of platelets further contributes to thrombocytopenia. Increased production of endothelial-derived platelet inhibitors prostacyclin and nitric oxide likely also contributes to impaired function of platelets (21).

This thrombocytopenia is compensated, however, by elevated levels of adhesive protein vWF and decreased ADAMTS-13 (Table 1). While vWF promotes platelet adhesion and aggregation at sites of vascular injury, it is negatively modulated by ADAMTS-13. ADAMTS-13 is a metalloproteinase that is primarily synthetized in the liver as well as in endothelial cells and platelets. It cleaves large vWF multimers into smaller less procoagulant forms and decreases clot formation. In acute liver failure vWF levels are elevated and ADAMTS-13 levels are reduced. These opposing effects promote platelet adhesion with the net result of rebalanced hemostasis (1, 22).

**Table 1:** Opposing effects of hemostasis in acute liver failure.

**Hemostasis**	**Anti-hemostatic**	**Pro-hemostatic**
Primary hemostasis: activated platelets and vessel wall interaction	↓Platelet Count ↓Platelet Function ↓Thrombopoietin ↑Nitric Oxide and Prostacyclin	↑ vWF ↓ ADAMTS-13
Secondary hemostasis/ coagulation: thrombin generation and inhibition	Low pro-coagulants factors ↓Factor II, V, VII, IX, X, XI, XIII ↓Fibrinogen Dysfibrinogenemia	Low anti-coagulant factors ↓Protein S and C ↓Antithrombin ↓Heparin Co factor ↑Factor VIII
Fibrinolysis: Fibrin removal	↓Plasmin Inhibitor ↓TAFI ↑t-PA	↓Plasminogen ↑PAI-1
Outcome	**BLEEDING**	**THROMBOSIS**
Treatment	Vit K, PCC, Factor VII, FFP, platelets, cryoprecipitate	Anticoagulation

*vWF, von Willebrand factor; ADAMTS-13, a disintegrin and metalloproteinase with a thrombospondin type 1 motif, member 13; t-PA, tissue plasminogen activator; TAFI, thrombin-activatable fibrinolysis inhibitor; PAI-1, plasminogen activator inhibitor type 1; PCC, Prothrombin complex concentrate*.

Thrombocytopenia is also compensated by the presence of platelet-derived extracellular vesicles (EV); microparticles (0.1–1 μm) which are highly pro-coagulant and contribute to ongoing activation of coagulation (23). Recently a study by Stravitz et al. showed that patients suffering from acute liver failure had an increased amount of these circulating microparticles (23, 24).

Overall, thrombocytopenia in ALF is compensated by elevated levels of vWF, reduced ADAMTS-13 levels and presence of platelet-derived extracellular vesicles that partially offset the impact of low platelet count.

### Secondary Hemostasis

Secondary hemostasis is characterized by the formation of an insoluble fibrin clot by activated coagulation factors and thrombin. Fibrin in turn stabilizes the primary platelet plug to stop the hemorrhage.

Reduction of coagulation factors is more extensive in acute liver failure compared to chronic liver failure, and factor levels can be as low as 1–10% of normal values (11, 21). Due to synthetic dysfunction of liver, there is reduced production of both pro-coagulant factors: V, VII, IX, X, XI, XIII, and prothrombin (II), and anti-coagulant factors: antithrombin, protein C, and protein S. Factor VIII is the only coagulation factor that is increased in PALF since Factor VIII is synthesized both in liver and in endothelial cells and is an acute phase reactant. In addition, activity of factor VIII is enhanced as a result of elevated levels of vWF, a protein that stabilizes Factor VIII in the circulation (25). These simultaneous and opposing pro- and anti-coagulant protein deficiencies results in a net rebalanced hemostasis.

Fibrinogen, an essential coagulation factor produced by the liver, it is the precursor of fibrin and is necessary for clot formation. Pediatric acute liver failure is accompanied by hypofibrinoginemia, though the reduction in fibrinogen levels is modest compared to the other pro-coagulant factors (2). Dysfibrinogenemia, or abnormal functioning of fibrinogen, is a result of increased content of sialic acid residues that leads to abnormalities of fibrin monomer polymerization (26, 27).

Vitamin K is essential for the synthesis of both pro-coagulant factors and anti-coagulant factors by the liver. Unlike in chronic liver failure, vitamin K deficiency is uncommon in PALF unless there is coexisting biliary tract disease or prolonged use of gut sterilizing broad-spectrum antibiotics. Deficiency has the potential to worsen the associated coagulopathy, and so differentiation between vitamin K-dependent and vitamin K non-dependent coagulopathy should be part of initial evaluation in PALF (28).

### Fibrinolysis

Fibrinolysis is the process by which the body breaks down clots. Fibrinolysis limits the extent of thrombosis, begins clot degradation, and maintains vascular patency. It starts with conversion of plasminogen to plasmin by the pro-fibrinolytic drivers tissue Plasminogen Activator (t-PA), urokinase-type Plasminogen Activator (u-PA), and activated factor XII. At the same time these pro-fibrinolytic drivers are opposed by anti-fibrinolytic drivers such as Plasminogen Activator Inhibitor type 1 (PAI-1), Plasmin inhibitor, and Thrombin-Activatable Fibrinolysis Inhibitor (TAFI) (1).

In liver disease, increased levels of t-PA and reduced levels of plasmin inhibitor and TAFI favors hyper-fibrinolysis and bleeding. On the other hand, reduced levels of plasminogen and increased levels of PAI-1 favors hypo-fibrinolysis and clot formation. These competing factors result in rebalanced pro- and anti-fibrinolytic activity in liver failure. In acute liver failure, high PAI-1 levels and low plasminogen levels may be substantial enough to tip the balance toward a hypofibrinolytic state whereas in cirrhosis, fibrinolysis is usually normal or hyper-fibrinolysis is present (2, 9, 21, 25).

Hyperfibrinolysis can also be seen during the anhepatic phase of liver transplantation due to increased tPA derived from endothelial cells; this is secondary to failure of tPA clearance in the absence of the liver (29).

Differentiating DIC and liver mediated coagulopathy can be quite challenging due to overlapping laboratory abnormalities and possibility of both coexisting at the same time. Patient with ALF may develop DIC if they become septic and hypotensive. Fibrinolysis of DIC is accompanied by fibrinogenolysis and by thrombin generation. Presence of fibrin-related markers such as D-dimer, Fibrin Degradation Products (FDPs) which is an important index of fibrinogenolysis may help differentiate true DIC from liver associated coagulation dysfunction (30–32). Decreased Factor VIII levels can also be used to distinguish DIC from hepatic disease in acquired hypofibrinogenemic states (33).

In summary, contrary to the common belief that acute liver failure patients are “autoanticoagulated,” they may be more prone to thrombosis rather than bleeding. There is a combination of (a) thrombocytopenia compensated with elevated levels of vWF, factor VIII and reduced ADAMTS-13, (b) a hypofibrinolytic state due to elevated PAI-1 and low plasminogen, and (c) elevated levels of pro-coagulant microparticles that may all lead to an increased risk of thrombosis and worsen outcome (23, 24).

## Limitation of Standard Hemostatic Tests

Current conventional laboratory tests such as PT/INR and partial thromboplastin time (PTT) have limitations for evaluating the coagulopathy of liver disease. Specifically, the PT/INR measures only procoagulant factors and omits the contribution of the platelets, *in vivo* inhibitors, fibrinolytic enzymes, and other cellular components. These tests do not provide information on actual fibrin formation or clot lysis. Lack of predictive power can be explained by the deficiency of anti-coagulant proteins which are reduced in parallel with pro-coagulant factors (34, 35).

The international normalization ratio (INR) is commonly used as a prognostic and decision-making tool when calculating the Pediatric End Stage Liver Disease Model (PELD) score which includes INR, bilirubin, albumin, and growth velocity of the child. International Normalized Ratio is a valuable biomarker for prognostication and determination of organ allocation; however it is inadequate for the measurement of bleeding risk. In children undergoing liver biopsy, hemorrhagic complications can occur in 0.91–4.2% of cases and coagulopathy markers do not predict bleeding complications (36, 37).

International Normalized Ratio calculation is derived from the Prothrombin Time (PT) and is standardized across laboratories among patients receiving vitamin K antagonists. Thrombomodulin is essential for PT measurement. Commercially available Prothrombin time tests do not contain a sufficient amount of thrombomodulin, and although the PT measures the function of pro-coagulants, it fails to measure the thrombin inhibited by the anticoagulants (1). Thrombin generation is downregulated by thrombomodulin, a transmembrane protein on endothelial cells which activates Protein C. Protein C is activated by thrombin and activated protein C forms a complex with its plasma co-factor, protein S and inhibits activated factor VIII and factor V that leads to less thrombin generation (Figure 1).

Furthermore, there is a significant laboratory to laboratory variation in the INR tests from patients with liver disease (10). One study demonstrated 26% variability in INR results among three laboratories, which could be problematic for treatment and organ allocation (3).

While standard assays of hemostasis are limited and misleading to evaluate hemostatic status in PALF, viscoelastic hemostatic assays (VHA) can accurately evaluate the current state of rebalanced hemostasis by measuring clot formation, ultimate clot strength, and the stability of the clot by integrating the contribution of platelets (8).

Viscoelastic hemostatic assays (VHA) such as thromboelastography (TEG) and rotational thromboelastometry (ROTEM) are whole blood tests that are a functional measure of clot formation and degradation in real time. Viscoelastic hemostatic assays measure the time to initial fibrin formation, the rate of clot formation, the strength of the clot, clot lysis and the contribution of sepsis-induced heparinoids to coagulation abnormalities. Since clot formation is the endpoint, VHAs have an obvious advantage over thrombin generation tests which have thrombin generation as the endpoint rather than formation of the fibrin clot (10, 14).

**Figure 2:**
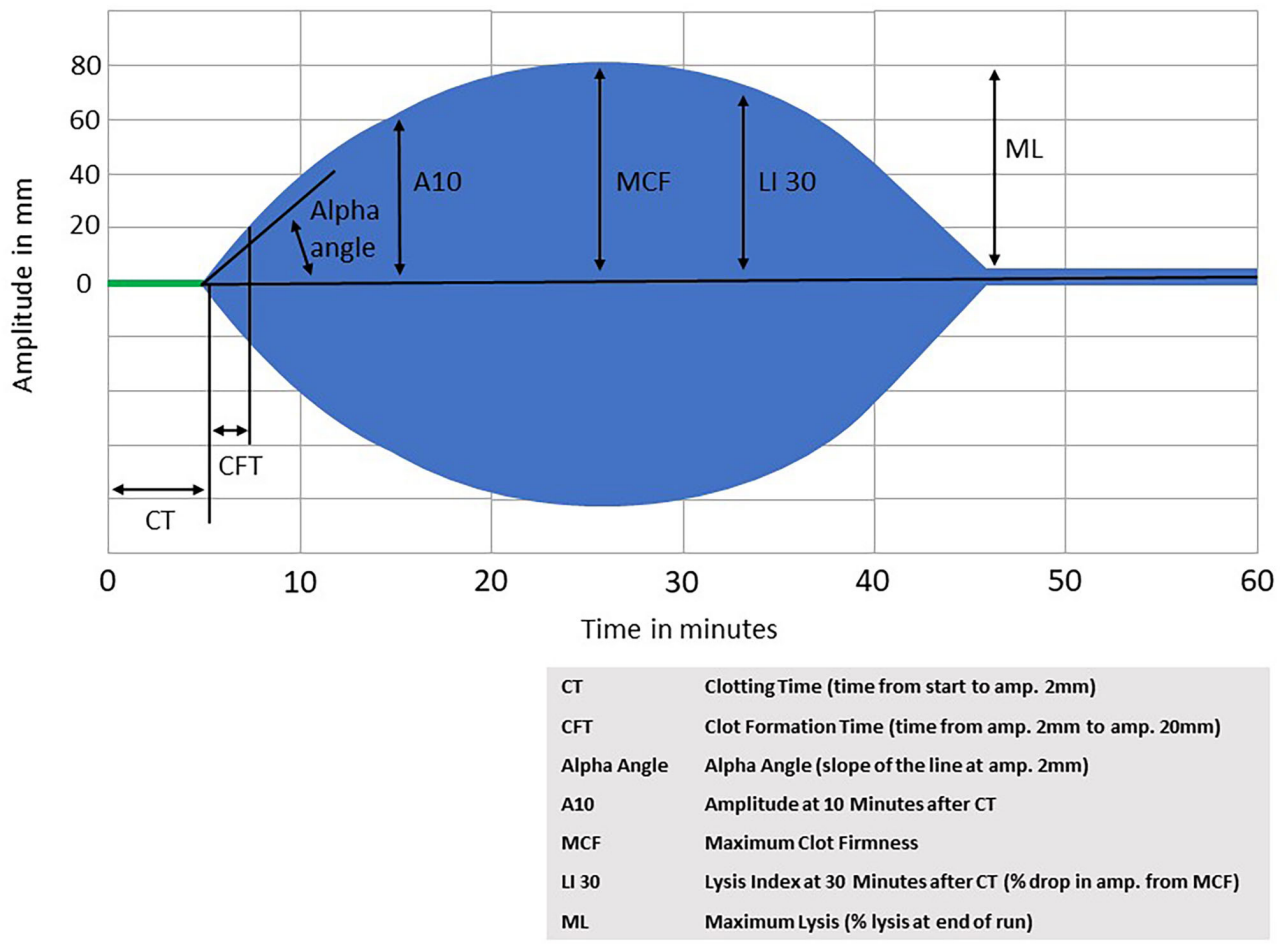
Rotational thromboelastometry (ROTEM).

Both TEG and ROTEM are commercially available point-of-care tests of whole blood coagulation. For TEG, blood is placed into an oscillating cup and treated with calcium and a kaolin-cephalin reagent. As the blood starts to clot, the viscoelasticity changes, and the blood starts to exert force on a pin that is suspended in the blood. The torque on the pin is converted into an electrical signal that results in a graphical representation of the clot formation and strength over time. As fibrinolysis begins and the clot starts to degrade, the force on the pin decreases and the resulting graph shows the clot dissolving. ROTEM is performed similarly, but with blood placed into a stationary cup, and an oscillating pin suspended into the blood sample. ROTEM also offers the possibility to use different activator reagents to assess different components of coagulation independently (such as the intrinsic pathway, extrinsic pathway, heparinization, fibrinogen contribution, and fibrinolysis. For an example of a ROTEM graph, see Figure 2.

Similar to patients with cirrhosis, patients with ALF generally have TEG parameters within normal limits. It has been shown that despite the average INR values of 3.4 (1.5–9.6) TEG parameters are within normal limits for adult ALF patients (8). Data on the use of VHAs specifically in pediatric patients with acute liver failure are lacking, and so one must extrapolate from the adult literature.

Hemostasis in children is an evolving process; there are age-dependent differences in the quantity and quality of hemostatic proteins that may further complicate the diagnosis of coagulopathy in children (29). A recent review summarized the published reference ranges for VHA testing in pediatrics and found that clotting times and clot formation times were shorter in healthy infants <6 months of age, suggesting more rapid initiation of clotting in this youngest pediatric age group (38). Generally, however, there were no significant differences found between children older than 6 months and adults in the parameters assessed by the assays, suggesting that assessment of clotting by VHAs is age-independent for children >6 months and adults. Utilization of VHAs in PALF patients may therefore provide a more accurate and reliable measure guiding transfusion practices and minimize unnecessary blood product administration.

## Therapeutic Implications

The coagulopathy and bleeding risks in acute and chronic liver failure are significantly different. Since portal hypertension is not significant in PALF and the thrombocytopenia is mild, bleeding risk is usually less compared to chronic liver disease (12).

There are no transfusion guidelines available for PALF and this leads to the substantial use of prophylactic blood products. There are no current pediatric cutoff values for the use of prophylactic transfusions of plasma, yet in adults an INR value of >7 with TEG confirming marked prolongation of clot formation, FFP transfusion is advised to maintain an INR between 5 and 7 (8). In PALF prophylactic correction of coagulopathy with fresh frozen plasma or thrombocytopenia with platelet administration is not recommended and should be avoided since the spontaneous bleeding complications are relatively uncommon (9, 12). In addition, INR trend, which is a commonly followed prognostic indicator in PALF, will be obscured with plasma administration. The currently available coagulation tests, other than VHA tests, do not measure the risk of bleeding accurately. Relying solely on those tests to guide transfusions will lead to patients being exposed to unnecessary blood products, leading to risk of fluid overload, transfusion-related acute lung injury (TRALI), and thrombosis risk (8).

Viscoelastic hemostatic assay tests, in contrast, may be able to provide the clinician with a more accurate assessment of coagulopathy. TEG and ROTEM have been used to develop resuscitation guidelines in adult trauma patients (39–41) and their use has been shown to decrease transfusions and mortality (41). Unfortunately there are currently no such guidelines published for either adult or pediatric acute liver failure patients. The use of VHAs in PALF would therefore require as astute clinician, trained in the interpretation of either TEG or ROTEM, in order to appropriately determine when interventions such as plasma, cryoprecipitate, platelets, or antifibrinolytics would be indicated for the pediatric patient with acute liver failure who is either at risk for, or currently bleeding.

Although it is generally mild, thrombocytopenia is the most significant contributor to the coagulopathic state in acute liver failure (8). Correction of platelet count and coagulopathy for high-risk procedures, such as liver biopsy and intracranial pressure (ICP) monitor placement, should be considered (18, 42). There are no cut-off values for preprocedural correction of coagulopathy in PALF, however in a recent survey among the participants of the Coagulation in Liver Disease Symposium, 50% of respondents suggested a cut-off value of INR > 1.5 and platelets of >30,000/mm^3^ for liver biopsy, and INR > 1.5 and platelet count of >50,000 mm^3^ for intracranial pressure monitor placement (18, 19, 42).

Vitamin K and fibrinogen deficiencies may prolong the INR/PT values and should be corrected as well. Oral or parenteral vitamin K should be considered if deficiency is suspected (34, 35). Cryoprecipitate may be used to keep fibrinogen in the low-normal range (150 mg/dl) and can be adjusted according to TEG values. Cryoprecipitate is a plasma-derived, concentrated product containing fibrinogen, Factor VIII, Factor XIII, vWF, and fibronectin. One unit of cryoprecipitate per 5 kg of body weight will increase fibrinogen by about 100 mg/dL. There are also plasma-derived fibrinogen concentrates available for the repletion of fibrinogen, but they are currently only approved for use in congenital fibrinogen deficiency and may not be available at all centers.

Pre-procedure correction of coagulopathy can be accomplished with low volume products such as Prothrombin Complex Concentrate (PCC) and recombinant activated factor VII (rFVIIa) (43). Prothrombin Complex Concentrate is a plasma-derived product containing clotting factors II, VII, IX, and X that is currently approved for reversing vitamin K antagonists. It has a low volume of administration and higher concentration of factors compared to plasma, which makes it an attractive alternative to FFP for factor repletion. There are several reports using PCC for patients with liver disease, some using a standard dose such as 25 units/kg, and others using an INR-based dosing regimen, similar to what would be used in vitamin K antagonist reversal (44). Caution is advised with administration of PCC given the lack of natural anticoagulants in this product compared to plasma, and the possibility of provoking thrombosis.

rFVIIa given at a dose of 20–40 mcg/kg, administered 30 min before the procedure has a rapid onset of action and a low volume of infusion making it another option for use prior to invasive procedures (42). However, administration of rFVIIa requires caution due to high cost and the risk of potentially serious thrombotic events (8, 45, 46).

Treatment goals in PALF should focus on acute bleeding management and prevention of infection, uremia, and GI bleeding, rather than prophylactic correction of laboratory values.

Antifibrinolytic agents such as aminocaproic acid and tranexamic acid (TXA) inhibit plasmin and prevent fibrin clot degradation. Although it has been shown that TXA may reduce blood loss during liver trauma or liver transplantation, there is no proven benefit for acute bleeding in PALF (46). These agents may be useful, however, in bleeding patients with documented hyperfibrinolysis seen on VHAs. These agents are frequently used in pediatric liver transplantation surgery at variable doses (29). A starting dose of TXA is typically 10 mg/kg given intravenously for bleeding. Similar to PCC and rFVIIa, caution should be taken with antifibrinolytics in patient with a history of thrombosis, and also any history of DIC or renal impairment.

Bacterial infections in ALF may potentiate the risk of bleeding, although the mechanism is speculative. Endotoxins and cytokines produced during infection may induce disseminated intravascular coagulation (DIC), inhibit platelet function, and enhance the effects of nitric oxide. Therefore, appropriate antibacterial prophylaxis and treatment of known infection is recommended (13).

Renal failure is also a common complication of acute liver failure. Uremia may further hinder primary hemostasis due to platelet dysfunction and impaired platelet-vessel wall interaction (47). Prevention and treatment of superimposed infections and renal failure are essential in order to prevent bleeding complications.

Clinically significant gastrointestinal bleeding is rare in PALF, however the American Association of Study of Liver Disease (AASLD) guidelines recommend histamine (H2) blockers or proton pump inhibitors for prophylaxis of gastric bleeding in ICU setting (8, 34, 48).

There are no guidelines for the resuscitation of acute bleeding in acute liver failure and transfusion of blood products have been associated with increased morbidity (48). Restrictive transfusion strategy with a threshold hemoglobin of >7 g/dL with a post-transfusion target of 7–9 g/dL are frequently utilized in a hemodynamically stable patient (15, 25, 42).

A catastrophic bleeding in PALF is very rare, most bleeding complications are clinically insignificant. Spontaneous bleeding usually due to self-limited upper gastrointestinal bleeding or post-procedural bleeding due to ICP placement (35). In case of catastrophic bleeding utilization of massive transfusion protocol may be warranted. Depending on patient laboratory values, a decisions can be made to give platelets, FFP, cryoprecipitate accordingly. If there is continues bleeding despite appropriate replacement of blood products, then PCC, of rFVIIa and TXA can be utilized.

## Summary and Future Directions

Hemostasis in pediatric acute liver failure exists in a rebalanced state due to concomitant changes in pro- and anti-coagulant mechanisms. This rebalanced hemostasis and lack of additional risk factors for bleeding (such as esophageal varices and portal hypertension that are commonly seen in chronic liver failure), leads to a low rate of spontaneous significant bleeding in PALF.

Current screening coagulation tests, such as the INR and PT are not predictive of acute bleeding and have certain limitations. International Normalized Ratio is useful for assessing severity of disease, however its use to evaluate one's bleeding risk is limited. Prophylactic FFP transfusion has not been shown reduce the risk of spontaneous bleeding and is not recommended in PALF. Arbitrary FFP and blood product transfusions may harm the patient with increased risks for fluid overload, cerebral edema, and elevated intracranial pressure. In addition, they will obscure the INR trend as a prognostic marker. Coagulopathy should be carefully evaluated and treated prior to high-risk procedures. Utilization of viscoelastic tests in PALF patients may provide an accurate assessment of bleeding risk, prevent unnecessary administration of blood products, lessen complications, and improve outcome. Further studies are necessary to characterize the coagulopathy in children with acute liver failure and develop treatment guidelines.

Understanding the pathophysiology of coagulopathy in PALF is important when evaluating pros and cons of transfusion and maintaining the delicate hemostatic balance. Etiology of PALF is different from adults, and there are age specific differences in pro-coagulant and anti-coagulant factors. It is important to maintain the fragile balance of hemostasis with restrictive transfusion strategies, and prevention of infection and uremia.

## Author Contributions

YB prepared the initial draft. GR and AS revised and edited the manuscript. All authors contributed to the article and approved the submitted version.

## Conflict of Interest

The authors declare that the research was conducted in the absence of any commercial or financial relationships that could be construed as a potential conflict of interest.
